# Early Life Adversity and Neuropsychiatric Disease: Differential Outcomes and Translational Relevance of Rodent Models

**DOI:** 10.3389/fnsys.2022.860847

**Published:** 2022-06-23

**Authors:** Renée C. Waters, Elizabeth Gould

**Affiliations:** Princeton Neuroscience Institute, Princeton University, Princeton, NJ, United States

**Keywords:** childhood maltreatment, anxiety disorders, major depressive disorder, PTSD, animal models, hippocampus, prefrontal cortex, amygdala

## Abstract

It is now well-established that early life adversity (ELA) predisposes individuals to develop several neuropsychiatric conditions, including anxiety disorders, and major depressive disorder. However, ELA is a very broad term, encompassing multiple types of negative childhood experiences, including physical, sexual and emotional abuse, physical and emotional neglect, as well as trauma associated with chronic illness, family separation, natural disasters, accidents, and witnessing a violent crime. Emerging literature suggests that in humans, different types of adverse experiences are more or less likely to produce susceptibilities to certain conditions that involve affective dysfunction. To investigate the driving mechanisms underlying the connection between experience and subsequent disease, neuroscientists have developed several rodent models of ELA, including pain exposure, maternal deprivation, and limited resources. These studies have also shown that different types of ELA paradigms produce different but somewhat overlapping behavioral phenotypes. In this review, we first investigate the types of ELA that may be driving different neuropsychiatric outcomes and brain changes in humans. We next evaluate whether rodent models of ELA can provide translationally relevant information regarding links between specific types of experience and changes in neural circuits underlying dysfunction.

## Introduction

Early life adversity (ELA) has long been recognized as a predisposing factor to the emergence of mental illness. While most ELA research has focused on childhood maltreatment, including abuse (physical, sexual, and emotional) and neglect (physical and emotional), observations that other types of childhood experiences can have deleterious effects on mental health have led to the recognition that ELA encompasses a broad range of negative experiences. Among these include exposure to natural disasters, war, chronic illness, death or incarceration of a caregiver, family separation, witnessing a violent crime, racism, and poverty. In this broad sense, ELA has been associated with an increased likelihood of developing several neuropsychiatric disorders, including, but not limited to, mood and anxiety disorders, posttraumatic stress disorder (PTSD), substance use disorders, and personality disorders (Widom et al., 1999; Bandelow et al., 2005; Scott et al., 2010; Lahti et al., 2012; Norman et al., 2012; Dagnino et al., 2020; Strathearn et al., 2020).

ELA also produces vulnerability to numerous physical conditions that are known to contribute to cognitive and emotional dysfunction, including excessive weight gain, diabetes, cardiovascular disease, autoimmune disorders, and certain types of cancer (Lippard and Nemeroff, 2020). Not surprisingly, given this long list of serious medical conditions, ELA has also been associated with shortened lifespan (Lippard and Nemeroff, 2020). Considerable research that investigates the connection between ELA and later life outcomes has considered ELA as a general umbrella term under which multiple conditions that produce chronic stress reside with the assumption that inherent differences in susceptibility, perhaps due to genetics, account for specific differences in health outcomes. However, a growing number of studies report evidence that certain types of ELA may produce specific vulnerabilities that lead to some, but not other, ELA-linked outcomes. This review considers a brief history of the study of the effects of ELA on negative health outcomes (with a focus on neuropsychiatric conditions). It then considers the degree to which variability in diagnostic outcomes can be explained by differential ELA experiences. In addition, the impact of different types of ELA on specific neural circuits, and whether these changes can be effectively modeled in experimental animals is discussed.

## The Link Between Early Life Adversity and Negative Health Outcomes: Historical Considerations

Although the impact of early childhood experience on the development of healthy mental processes in adulthood was acknowledged in Western society as early as the eighteenth century (Gianoutsos, 2006), it was not until the late nineteenth century that child abuse was recognized as a criminal offense in the US and resources were put into protecting children (Crosson-Tower, 2021). Although physical maltreatment (abuse and neglect) during childhood was considered to be inhumane and potentially harmful to long-term health outcomes, the link to mental health was not reported in the scientific literature until the 1960s when “battered child syndrome,” which includes signs of what would now be referred to as major depressive disorder (MDD) and PTSD, was characterized (Kempe et al., 1962; Galdston, 1965), and shown to have lasting impacts on psychiatric health into adulthood (Isaacs, 1968). It was later recognized that childhood sexual abuse increases the likelihood of anxiety disorders, MDD, suicidality, and substance use disorder (Tsai and Wagner, 1978; Rosenfeld, 1979). Subsequently, scientific reports showed that increased incidence of psychiatric disorders has been linked to verbal abuse (Zanarini et al., 1989; Kjelsberg et al., 1994).

Around the same time that reports of physical and sexual abuse increasing susceptibility to mental illness emerged, studies showed that emotional neglect in childhood is also a risk factor for clinical depression (Jacobson et al., 1975), with additional studies reporting that loss of the primary caregiver, typically the mother, which often leads to emotional neglect, has a similar effect (Goldney, 1981; Harris et al., 1986; Patten, 1991).

Although many of the earlier studies presented compelling evidence for links between childhood adversity and mental illness, most included relatively small samples and used basic metrics for determining whether abuse or neglect occurred. To address these issues and to determine the overall health outcomes of individuals subjected to childhood adversity, a landmark study was done by the Center for Diseases Control (CDC) and Kaiser-Permanente beginning in 1995 (Felitti et al., 1998). This study used family health history and health appraisal questionnaires that collected a large amount of information from over 13,000 adults living in the US about their childhood experiences and health (both mental and physical). The questionnaire included three ELA categories, each with distinct subtypes: abuse (physical, emotional, sexual), neglect (physical, emotional), and household dysfunction (mental illness, incarcerated relatives, mother treated violently, substance abuse, and parental divorce). The study presented strong evidence that a high degree of childhood adversity increased the risk of clinical depression, suicidality, and substance use disorder, as well as of physical conditions that are often comorbid with psychiatric diagnoses, including obesity, cardiovascular disease, diabetes, and cancer (Felitti et al., 1998). This study also presented evidence for a “dose-response” relationship between childhood adversity and health outcomes whereby a greater number of adversities was statistically correlated with a higher risk for certain illnesses. Since the publication of this report, numerous studies have used similar questionnaires. These studies have firmly established the relationship between ELA and mental illness and allowed for estimates by the World Health Organization (WHO) that ELA accounts for about thirty percent of all psychiatric disorders (Kessler et al., 2010). However, even with the broad range of experiences considered to be ELA in these studies, they do not capture the diversity of adversity experienced by the global population or even within the United States. More recent studies have added to the list of adverse childhood experiences that increase the risk of health problems, such as bullying by peers (Biedermann et al., 2021; Iob et al., 2021), being involved in an environmental disaster (Adebäck et al., 2018), being treated for a chronic illness (Bergmans and Smith, 2021), being subjected to family separation due to parental incarceration within countries (Murray and Farrington, 2008; Swisher and Shaw-Smith, 2015) and at the US-Mexico border (Teicher, 2018; de la Peña et al., 2019; MacLean et al., 2020; Sidamon-Eristoff et al., 2022), economic hardship (Mawson and Gaysina, 2021), neighborhood violence (van Rooij et al., 2020), and racism (Thomas Tobin and Moody, 2021).

## Variation in Deleterious Effects of Early Life Adversity

Although the link between ELA and increased vulnerability to illness has been well-established through earlier studies, the high variability in individual health outcomes is notable. A sizable proportion of individuals exposed to ELA do not seem to differ from the non-ELA population in terms of risk for developing mental health problems. This resilience has been attributed to possible genetic or mitigating environmental factors (Ensink et al., 2021). Even within the affected population of ELA-exposed individuals, considerable variability in the type of psychiatric illness, as well as the types of symptoms exhibited within the diagnostic criteria, exists in the outcome. Studies have considered how different onset, severity, and duration of ELA experiences contribute to much of the variability. Many findings align with the idea that earlier occurrences of maltreatment and poverty are associated with greater stress-related brain changes (Andersen and Teicher, 2008) and mental health issues in adulthood (Fitzsimons et al., 2017). Theorists who prioritize timing in models of the impact of ELA often base their predictions on “experience-expectant” mechanisms of neurodevelopment, where adversity during sensitive or critical periods is more likely to have profound effects on neural function in adulthood (Nelson and Gabard-Durnam, 2020).

It is also relevant to mention that gender is a significant mediator between ELA and later life neuropsychiatric outcomes (Thompson et al., 2004; Fisher et al., 2009; Fletcher, 2009; Meng and D’Arcy, 2016). These gender differences may be attributed to differences in the adverse experience itself, in terms of frequency and/or intensity (Thompson et al., 2004; Maikovich-Fong and Jaffee, 2010; Meng and D’Arcy, 2016; Gauthier-Duchesne et al., 2017; Giano et al., 2020; Khan et al., 2021). It is also probable that the differences between these two populations are reflective of differences in reporting rates between sexes. Males are less likely to report sexual abuse and interpersonal violence, while they are more likely to report physical and emotional abuse (Meng and D’Arcy, 2016). Gender differences in the relationship between ELA and neuropsychiatric outcomes may also be attributed to reduced sample sizes in male participants due to fewer seeking professional help for mental health issues (Pattyn et al., 2015). Lastly, and most relevant to the current review, during development there are likely significant differences in the developmental trajectories of stress-sensitive circuits between sexes. Consequently, ELA may impact male and female youth differently, ultimately contributing to differences in neuropsychiatric outcomes (Hill et al., 2010; Dragan et al., 2019; Bath, 2020).

Studies show that less than five percent of childhood adversity occurs as a single event. Not only do people often experience multiple types of ELA but the cumulative effects of these different types of ELA have proven to be deleterious (Dich et al., 2015). In the CDC/Kaiser-Permanente study discussed above, researchers found that nearly one in five children had experienced two or more types of ELA. Researchers have found that the combination and accumulation of ELA increase the severity of neuropsychiatric diseases across multiple psychiatric diagnoses, including MDD and anxiety disorders (Anda et al., 2006). Taken together, studies that have considered whether timing or severity determines the qualitative outcome have concluded that although these factors play some role, they do not account for all the variability.

The unexplained variability raises the possibility that specific types of childhood experiences change the brain in ways that lead to disparate negative health outcomes. Although some of the earliest reports linking childhood experiences with mental health outcomes have attempted to draw connections between specific experiences and specific symptoms (Bornstein, 2006), the field has moved increasingly toward considering ELA experiences as having common stress consequences that produce different outcomes because of factors inherent to the individual. Recently, however, a growing literature has investigated whether specific adverse experiences are major determinants of symptom variability. In the next section, different categories of ELA are considered in the context of neuropsychiatric correlates, as well as what is known about related neural changes. As mentioned previously, childhood maltreatment rarely occurs in an isolated form (Felitti et al., 1998), however, it is important to consider evidence suggesting that there may be specific contributions of each subtype to the etiology of some neuropsychiatric illnesses. It should be noted at the outset of this discussion that there is considerable overlap in conditions that have been associated with specific categories of ELA and that some studies have reported that only non-specific relationships exist, including that there are no major differences in brain effects (Haidl et al., 2021). Because the majority of research on this topic has considered forms of ELA that involve childhood maltreatment, the following discussion will focus on physical abuse, sexual abuse, and emotional maltreatment.

## Physical Abuse

Despite numerous societal efforts to reduce its occurrence, childhood physical abuse remains common (CDC, 2021). The field seems to generally conclude that individuals who experience physical abuse are at an increased risk of developing anxiety disorders, PTSD, and certain personality disorders, including antisocial and borderline (Kiser et al., 1991; Bornovalova et al., 2006; Springer et al., 2007; Fergusson et al., 2008; Schorr et al., 2020). Perhaps not surprisingly, the mental health risks for children who experience both physical and sexual abuse are potentiated (Roth et al., 1997). When studies have attempted to dissect these findings, such as to determine which abuse subtype is the strongest predictor, the evidence seems to differ considerably, possibly because most studies have not considered other factors, such as duration, severity, or onset. A recent study attempted to fill this gap by examining 1,200 emerging adults and found that physical abuse beginning between 6 and 12 years old is associated with several types of psychopathologies but that physical abuse at age 13 or older was a significant predictor of mainly PTSD (Adams et al., 2018).

**FIGURE 1:**
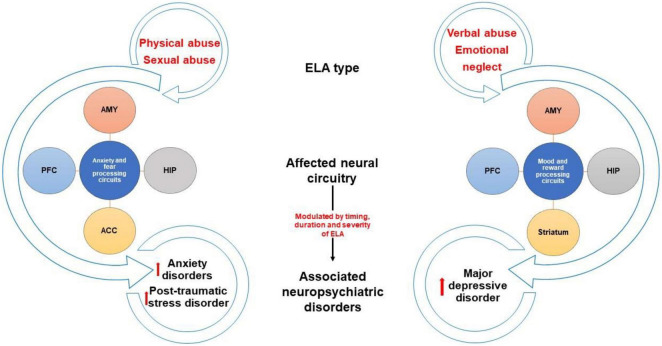
Brain changes and neuropsychiatric outcomes associated with different ELA types. Diagram depicting brain regions that are altered by different types of childhood maltreatment. Studies show that physical and sexual abuse can alter regions involved in anxiety/fear processing circuits while verbal abuse and emotional neglect affect regions related to mood/reward processing circuits. These brain changes likely contribute to differential predisposition to certain neuropsychiatric disorders, such as anxiety disorders, post-traumatic stress disorder, and major depressive disorder. ACC, anterior cingulate cortex; AMY, amygdala, HIP, hippocampus, PFC, prefrontal cortex.

These findings raise questions about how physical abuse might change the brain to increase the likelihood of anxiety disorders and PTSD. Studies have shown that compared to healthy controls, physically abused children have smaller amygdala, prefrontal cortex, and hippocampal volumes (Gold et al., 2016; Lambert et al., 2017; Nogovitsyn et al., 2020), as well as altered activation in brain regions involved in emotional regulation and fear processing, including greater activation of the amygdala, hippocampus, and medial prefrontal cortex when presented with emotional faces (Garrett et al., 2012; McLaughlin et al., 2019; Table 1). These studies suggest that the lasting effects of physical abuse may be the result of changes in emotion and fear processing circuits brought about by threat-induced activation of these brain regions during sensitive periods of development (Figure 1).

**TABLE 1:** Brain changes and neuropsychiatric disorders associated with different types of ELA in humans.

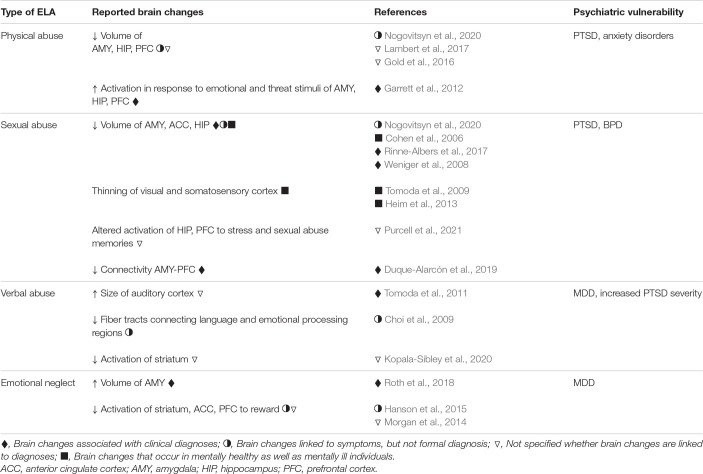

## Sexual Abuse

There is considerable overlap between the most common mental health outcomes associated with sexual abuse and those associated with physical abuse, although the relationship between sexual abuse and increased susceptibility to PTSD and borderline personality disorder seems especially strong (de Aquino Ferreira et al., 2018; Hailes et al., 2019; Breuer et al., 2020; Cancilliere et al., 2021). In addition, studies suggest that sexual abuse leads to increased dissociative symptoms (Tschoeke et al., 2021) and non-suicidal self-injury and suicidal ideation, but not completion (Mullen et al., 1994; de Aquino Ferreira et al., 2018). The latter effects seem to be magnified when sexual abuse was combined with physical abuse, as well as when the abuse was repeated and occurred over a long time (de Aquino Ferreira et al., 2018). Aside from diagnostic outcomes, sexual abuse has been linked to interpreting neutral facial expressions as negative (Pfaltz et al., 2019) and to deficits in social cognition (Duque-Alarcón et al., 2019) and other cognitive functions (Navalta et al., 2006) as well as impaired parenting skills (Burkett, 1991; Cole et al., 1992; Ethier et al., 1995).

Several studies suggest that childhood sexual abuse changes the brain in ways that are not necessarily specific to the type of ELA or the presence or absence of psychopathology. Childhood sexual and physical abuse have been linked to reduced volume of the amygdala regardless of clinical diagnosis (Nogovitsyn et al., 2020) suggesting that early exposure to trauma alters the structural development of this region, perhaps contributing to vulnerability to subsequent stress. Childhood sexual, but not physical, abuse has been associated with diminished stress-induced activation of the dorsolateral frontal cortex and hippocampus (Purcell et al., 2021). Childhood sexual abuse produces thinning of the visual cortex and somatosensory cortex associated with genital representations in a way that may not be related to the development of psychopathology as the effects can be observed even in individuals without diagnoses (Tomoda et al., 2009; Heim et al., 2013). Additional brain changes have been observed in individuals with a history of childhood sexual abuse presenting with a psychiatric diagnosis. For example, people with a history of childhood sexual abuse and a PTSD diagnosis exhibit greater activation of the prefrontal cortex in response to childhood sexual abuse memories (Bremner et al., 1999), a shift in activation from the frontal cortex to the hippocampus and amygdala when viewing emotional faces (Duque-Alarcón et al., 2019), as well as diminished volume of the anterior cingulate (Cohen et al., 2006; Rinne-Albers et al., 2017). Childhood sexual abuse victims with a BPD diagnosis have diminished connectivity between the amygdala and the frontal cortex (Duque-Alarcón et al., 2019; Table 1). These results suggest that sexual abuse likely has general as well as specific effects on the brain, some of which may be protective and others that contribute to vulnerability, perhaps in cases where the individual experiences additional adversities during development or in adulthood. Differences in the size of key brain regions as well as in the recruitment of specific circuitry in response to subsequent stress may explain the increased likelihood for childhood sexual abuse victims to develop certain symptoms, particularly those related to emotional processing (Figure 1).

## Emotional Maltreatment

While physical and sexual abuse both evoke threat of bodily harm, emotional maltreatment involves threat to self-image. In addition, emotional maltreatment often involves either direct or indirect emotional deprivation, although studies have shown that even in cases where emotional abuse does not involve net emotional deprivation, the effects are negative. For example, emotional abuse has been strongly linked to MDD, with studies showing that its effects are not mitigated by emotional affection by the abusive or other parent (Polcari et al., 2014). It should be noted that some studies have shown that childhood physical and sexual abuse have been linked to MDD (Lansford et al., 2002; Maniglio, 2009), but the relationship between emotional abuse and neglect appears to be stronger (Gibb et al., 2001; Kim and Cicchetti, 2006; Martins et al., 2014; Infurna et al., 2016), and, of course, it is important to consider that physical and sexual abuse are often accompanied by verbal abuse, making it difficult to isolate the effects of these types of ELA separately. Related to the impact of parental emotional abuse on the later emergence of psychopathology, many recent studies have shown a link between bullying by peers and the development of MDD in adulthood (Biedermann et al., 2021; Iob et al., 2021; Valera-Pozo et al., 2021), raising the possibility that similar mechanisms are invoked by emotional abuse from caregivers as from peers. In addition to increasing the likelihood of MDD, emotional abuse during childhood has been linked to the severity of PTSD (Rameckers et al., 2021), which is often comorbid with MDD.

As mentioned earlier, emotional abuse, particularly when delivered by a parent, is often accompanied by emotional neglect, so it is particularly difficult to study the latter alone. However, studies of children raised by mothers with postpartum depression, who are therefore less interactive, as well as those who lose their primary caregiver as a result of death or incarceration may provide some insight. These studies have shown an increased incidence of MDD (Berg et al., 2016; Netsi et al., 2018; Heard-Garris et al., 2019) with the timing of parental loss as a mediating factor (Berg et al., 2016).

There are several aspects of emotional abuse and neglect that may contribute to its strong ties to depressive disorders. A long line of developmental research supports the view that attachment figures are critical for the development of healthy social schemas of the world (Amos et al., 2011; Szepsenwol and Simpson, 2019). Emotional neglect deprives the child of necessary prosocial experiences, while emotional abuse shifts social interactions in a negative direction, increasing the likelihood of developing a “negative self-model,” which often leads to depressive illness (Infurna et al., 2016). Emotional abuse in childhood also has a significant impact on social function in adulthood by increasing the likelihood of fear and avoidance of social situations. Since positive social relationships can buffer against subsequent stress and social avoidance is often a feature of MDD, it is reasonable to assume that these changes are related.

Given the long-term impact of emotional maltreatment, studies have sought to determine how the brain is changed by these experiences. Emotional maltreatment in general has been shown to increase activation of the amygdala (Puetz et al., 2020). Verbal abuse in particular, has been shown to increase the size of parts of the auditory cortex (Tomoda et al., 2011), as well as diminish fiber tracts connecting language and emotional processing regions (Choi et al., 2009; Tomoda et al., 2011; Table 1). These findings suggest that persistent activation of auditory and language processing regions, particularly under conditions of stress, alter the developmental trajectory of these circuits, likely affecting how subsequent verbal information is processed. Emotional neglect has been shown to produce not only a larger amygdala volume (Roth et al., 2018) but reduced volume and altered function in a frontoparietal network that has been associated with complex social and cognitive processing (McLaughlin et al., 2019; Puetz et al., 2020; Table 1).

These findings suggest that emotional maltreatment may alter development of circuits that are critical for processing salient socioemotional information in adulthood. Evidence also suggests that emotional maltreatment produces changes in reward circuitry, including activation of the prefrontal cortex and striatum in response to reward (Morgan et al., 2014; Hanson et al., 2015; Kopala-Sibley et al., 2020). This dysregulation may contribute to the emergence of mood disorders, of which reward processing deficits can be defining symptoms. It should be noted that some research has shown that sex differences exist in the extent to which brain regions are impacted by emotional maltreatment, findings that may be related to sex differences in symptoms, as well as in rates of MDD and anxiety disorders (Altemus et al., 2014).

## Remaining Questions About Specific Impacts of Early Life Adversity Subtypes in Humans

Given that physical abuse, sexual abuse, verbal abuse, and emotional deprivation have been shown to predispose to different, albeit overlapping, neuropsychiatric conditions, it seems reasonable to consider not only whether psychiatric patients have histories of ELA, but the individual specific life history when developing treatment plans. Despite this somewhat obvious conclusion, it remains unknown whether a psychiatric condition, e.g., MDD, that arises from one type of ELA, e.g., verbal abuse, has the same or different underlying neural dysfunction as that which arises from another type of ELA, e.g., parental loss. While talk therapy can address childhood experiences directly, biomedical interventions may benefit from information about specific experiential etiologies, particularly if it is found that they involve differing brain changes. It seems relevant to note that although multiple types of antidepressant treatments exist, it remains unclear which treatments will be most effective for individual patients, and 10–30% of patients with MDD either show only a partial improvement or appear to be resistant to all treatments (Al-Harbi, 2012). Similar issues exist regarding the inability of health care professionals to alleviate suffering in many people with PTSD and BPD, conditions that are typically treated with drugs developed for other conditions, like MDD and anxiety disorders. A more informed approach toward treating such conditions in a way that is tailored to the individual patient can be envisioned to arise from a better understanding of how specific forms of ELA impact the brain differentially. Even though the field has progressed considerably by recognizing that different subtypes of ELA predispose more heavily to certain psychiatric conditions than others, and that ELA subtype-specific brain changes are known to occur, there remain considerable unanswered questions about how ELA impacts neural mechanisms that might serve as potential therapeutic targets.

## Modeling Early Life Adversity in Experimental Animals

To study the neurobiological consequences of ELA, scientists have developed models using experimental animals to test causal mechanisms and investigate levels of analyses that are not possible to probe in humans. While all of the types of ELA experienced by humans cannot be captured in these models, the expectation has been that this approach will provide some insight into how ELA impacts molecular and cellular processes in specific neural circuits, so that the information can be used to build a preclinical framework that would be useful for treating humans suffering from ELA-related conditions. Although some non-human primate models of ELA exist, practical and ethical issues have limited their usefulness, and most of the research on this topic has used rodents (Murthy and Gould, 2018). For rodent experiments to effectively model ELA-induced predispositions to psychopathology seen in humans, they should produce behavioral phenotypes that mimic some aspects of the relevant disorders. This is a difficult problem to solve since neuropsychiatric diseases linked to ELA in humans, including MDD, anxiety disorders, PTSD, and personality disorders, cannot be replicated in rodents without resorting to debatable anthropomorphic interpretations of animal behavior (LeDoux and Pine, 2016; Murthy and Gould, 2020). Despite this important caveat in interpreting behavioral data from these studies, several ELA rodent models have emerged that produced interesting behavioral results pointing to the potential for modeling differential effects in humans. They are described below grouped into the types of human ELA they might represent, with the recognition that rodent experiences are likely very different from human ones, even when they share some common surface features.

A comprehensive understanding of how ELA affects the brain and behavior requires consideration of potential sex differences. In humans, there are gender differences in the prevalence of exposure to adversity (e.g., girls are more likely than boys to be exposed to sexual abuse; boys are more likely than girls to be exposed to non-sexual physical abuse, Tourigny et al., 2008; Lippus et al., 2020), as well as in diagnoses of neuropsychiatric disorders linked to ELA (e.g., women are more likely than men to be diagnosed with anxiety disorders or major depressive disorder; men are more likely to be diagnosed with substance use disorder, Altemus et al., 2014; NIDA, 2021), and societal influences are difficult to rule out. ELA studies in experimental animals have traditionally included only male rodents, but a growing number of studies have considered both sexes with a view toward identifying potential sex differences (Bath, 2020). In the next section, rodent sex difference findings are incorporated into our discussion of the effects of specific rodent ELA paradigms.

It should be noted that prenatal stress has also been shown to impact behavior of offspring in ways that may reflect aspects of neuropsychiatric disease in humans (reviewed in Weinstock, 2016), but since here we are focused on modeling adversity during infancy, childhood, and adolescence, our discussion will consider only studies that include manipulations during postnatal life.

## Physical Pain

Some researchers have investigated the impact of painful physical experiences during development on rodent behavioral outcomes in adulthood. Since these manipulations impact somatosensory pain pathways and directly signal threats to safety, they may share common outcomes with physical abuse in children. Studies have used unpredictable electric shock in neonates and adolescents, formalin injection, or paw surgery to examine behavioral outcomes and found increased avoidance behavior (Negrigo et al., 2011; Sarro et al., 2014; Li et al., 2015; Tao et al., 2017; Lovelock and Deak, 2019; Ririe et al., 2021; Zhao et al., 2022) and enhanced fear conditioning (Quinn et al., 2014) in rats. Increased avoidance behavior has been observed in both male and female rats treated with formalin injection, with greater effects observed in females (Negrigo et al., 2011). Footshock during adolescence has been shown to increase avoidance behavior in male but not female rats (Lovelock and Deak, 2019), however another study showed increased avoidance behavior in female rats (Tao et al., 2017). The reasons for these sex difference discrepancies remain unknown but could be due to different timing and duration of stress exposure.

Increased avoidance and enhanced fear conditioning with models of early life physical abuse may be consistent with the most common neuropsychiatric disorders stemming from physical abuse in humans, namely, PTSD and anxiety disorders. Likewise, brain data obtained from these models is also consistent with what has been observed in humans subjected to childhood and adolescent physical abuse, such as changes in the amygdala (Sarro et al., 2014), including increased activation (Lovelock and Deak, 2019; compare Table 2 with Table 1).

**TABLE 2:** Brain changes and behaviors resulting from different types of ELA in rodents.

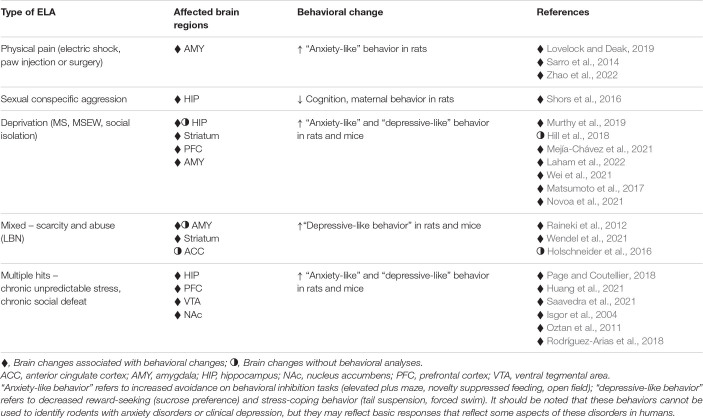

## Sexual Aggression

It is particularly difficult to model childhood sexual abuse in laboratory rodents because mating activity depends on hormonal as well as behavioral sexual receptivity, which only emerges after puberty. Shors et al. (2016) developed a novel model of repeated sexual abuse whereby adolescent female rats were repeatedly paired with large sexually experienced male breeders, which produced chasing, fighting, and mating attempts. Because the females were not through puberty, and as such, were not behaviorally sexually receptive, there were no completed intromissions, but these experiences were stressful for the females in that they produced activation of the HPA axis (Shors et al., 2016). This paradigm was not examined for effects on avoidance behavior, but the researchers found that female rats exposed to sexually aggressive males exhibited diminished cognitive ability, as well as reductions in the maternal care of unrelated pups (Shors et al., 2016). The extent to which these rodent behavioral changes reflect those observed in victims of early sexual abuse in humans remains unknown, but it may be relevant that childhood sexual abuse has been associated with reduced cognitive function (Navalta et al., 2006) as well as impaired parenting skills (Burkett, 1991; Cole et al., 1992; Ethier et al., 1995). Very limited evidence exists regarding the effects of this paradigm on the rodent brain, but available data suggest a reduction in neuronal growth in the hippocampus (Shors et al., 2016), which is consistent with work in humans showing that hippocampal volume and activation are reduced after childhood sexual abuse (Weniger et al., 2008; Yuan et al., 2020; Purcell et al., 2021; compare Table 2 with Table 1). Clearly, this approach presents an innovative experimental animal paradigm that should be explored further for potential translational relevance. To our knowledge, there are no available animal models of sexual abuse in male rodents, so examination of sex differences is not possible.

## Emotional and Physical Deprivation

The first ELA model to be developed for rodents, a model of deprivation called maternal separation (Ader et al., 1960), remains the most used to this day. This paradigm involves separating pups from their dam for varying amounts of time (usually for 1–8 h daily) during the first 2 postnatal weeks. Despite some attempts to standardize the separation times, the behavioral results of this manipulation vary considerably. Several studies have discussed how maternal separation has differential outcomes regarding behaviors that have been considered “anxiety-like” and “depressive-like” (Pryce and Feldon, 2003; Millstein and Holmes, 2007). Systematic reviews and meta-analyses of the literature regarding maternal separation show that there are no consistent increases in avoidance behavior following maternal separation (Tractenberg et al., 2016; Murthy and Gould, 2020; Wang et al., 2020). This is not surprising given the variability in response to ELA due to sex (Bath, 2020), genotype (Holmes et al., 2005; Millstein and Holmes, 2007; Mehta and Schmauss, 2011), and maternal separation procedure (Lehmann and Feldon, 2000; Wang et al., 2020). While there is no consensus on how maternal separation impacts avoidance behavior, there is a stronger case for the connection between maternal separation and “depressive-like” behavior in mice (Tractenberg et al., 2016). Despite differences in strain, species, and protocol, many studies agree that maternal separation leads to an increase in “depressive-like behavior,” defined as reduced stress coping in the forced swim test (FST) and tail suspension test (TST), as well as reduced reward seeking in a sucrose preference test in mice and rats (e.g., Tchenio et al., 2017; He et al., 2020; Thornton et al., 2021; Wei et al., 2021). As with the physical stress/pain studies, maternal separation studies have shown varied differences with sex; some studies show effects on males with no effects on females (Demaestri et al., 2020; He et al., 2020), others show effects in females and not males (Honeycutt et al., 2020), while still others show comparable effects on both sexes (Thornton et al., 2021). The reasons for these discrepant results remain unknown but could be due to differences in species (mouse, He et al., 2020; rat, Honeycutt et al., 2020; Thornton et al., 2021), in durations of maternal separation per day (6 h per day, Thornton et al., 2021; 4 h per day, He et al., 2020; Honeycutt et al., 2020), or different durations of maternal separation overall (P2-P12, He et al., 2020; P2-P20, Honeycutt et al., 2020; P1–P21, Thornton et al., 2021).

A newer model than maternal separation alone, maternal separation and early weaning, which may be a more cumulative model of ELA, has produced results that seem to show increased “depressive-like” behavior (George et al., 2010; Montalvo-Ortiz et al., 2016; Sun et al., 2021) as well as increased behavioral avoidance (George et al., 2010; Carlyle et al., 2012; Murthy et al., 2019; Sun et al., 2021), perhaps reflecting the high comorbidity between MDD and anxiety disorders, but there are still reports showing inconsistent results (Tan et al., 2017; Waters et al., 2022). Maternal separation and early weaning studies have generally shown effects in male mice and rats (George et al., 2010; Montalvo-Ortiz et al., 2016; Murthy et al., 2019; Sun et al., 2021), with studies either not examining females or, if so, finding no effects (Murthy et al., 2019; Sun et al., 2021). The lack of effects in females may be due to the protective action of certain stages of the estrous cycle in that maternal separation and early weaning mice only shows increased avoidance behavior during diestrus, a time when the ratio of progesterone to estrogen is relatively high (Laham et al., 2022). These findings raise possible connections with human studies showing that childhood maltreatment increases the likelihood of emotional dysfunction linked to fluctuations in ovarian hormones (Azoulay et al., 2020).

Aside from depriving preweaning pups of maternal contact, additional studies have examined deprivation in adolescence by using models of social isolation housing after weaning. These studies have shown that social isolation reduces stress coping, social interaction, and reward seeking, behaviors that may reflect “depressive-like” and “anxiety-like” phenotypes (Chang et al., 2015; Matsumoto et al., 2017; Novoa et al., 2021; Potrebić et al., 2021).

Studies involving maternal separation during the preweaning period or social isolation during adolescence have revealed brain changes in areas that have been shown to be affected in humans subjected to early life emotional neglect, such as the medial prefrontal cortex, hippocampus, amygdala, and striatum (Edmiston et al., 2011; Morgan et al., 2014; Matsumoto et al., 2017; Hill et al., 2018; Murthy et al., 2019; Peña et al., 2019; Mejía-Chávez et al., 2021; Novoa et al., 2021; Laham et al., 2022). The extent to which the reported brain changes after maternal separation are similar to those in humans subjected to emotional neglect (compare Table 2 with Table 1), beyond the commonalities in the affected brain regions remains unknown.

## Mixed and Multiple Hit Models

The limited bedding and nesting (LBN) model is now widely used as an ELA paradigm. LBN involves reducing or eliminating bedding material and reducing nesting material available to the dam. LBN is thought to model scarce resources in childhood that can lead to erratic and potentially abusive maternal care (Gallo et al., 2019), so this model may reflect a combination of environmentally induced neglect and abuse in humans. However, it should be noted that LBN, although reported to increase aggressive behavior in some dams toward their pups (Gallo et al., 2019), has also been shown to increase nursing and attentive care, perhaps as a compensatory response (Eck et al., 2020), so its impact on offspring may vary dramatically depending on individual differences in maternal response. Similar to maternal separation studies, experiments using LBN have resulted in varied behavioral outcomes related to “anxiety-” and “depressive-like” behavior in adulthood with some studies showing increases (Dalle Molle et al., 2012; Raineki et al., 2012; Wendel et al., 2021) and others showing decreases or no change (van der Kooij et al., 2015; Johnson et al., 2018). Regarding sex differences, seemingly discrepant results from LBN rearing have been reported. For example, one study showed LBN-induced increased avoidance behavior in males but not females (Dalle Molle et al., 2012) while another showed no changes in avoidance behavior in either sex but reduced stress coping and reward seeking in females (Goodwill et al., 2019). The reasons for these differences could be related to the species (rat, Dalle Molle et al., 2012; mouse, Goodwill et al., 2019) or timing of LBN (P2-P9, Dalle Molle et al., 2012; P4-P11, Goodwill et al., 2019). In studies where increases in “anxiety-like” and “depressive-like” behaviors have been observed, associated brain changes point to alterations in regions linked to avoidance and fear processing circuitry, including the amygdala (Raineki et al., 2012) and anterior cingulate (Holschneider et al., 2016), as well as reward circuitry, including the ventral tegmental area and nucleus accumbens (Wendel et al., 2021; Table 2).

Rodent models have also been developed to simulate the unpredictable, cumulative effects of ELA that often occur throughout childhood and adolescence in humans. By exposing rodents to repeated exposure to the same stressor and/or by combining exposure to different types of stressors over the developmental period, recovery and/or adaptation to adversity may be prevented. These models may be more naturalistic and reflective of the human experience, where adversity is often diverse and repeated, and have been generally successful at inducing behavioral changes (Molet et al., 2014). “Multiple-hit” models during development have been shown to have a significantly greater impact than a single adversity alone (Hollis et al., 2013).

A relevant animal model of the impact of repeated emotional and physical abuse may be found with studies investigating repeated social defeat during adolescence. This paradigm typically involves a brief episode of physical contact with an aggressive conspecific, after which the aggressor and test animal are separated by a physical barrier that permits repeated visual, auditory and olfactory contact without the possibility of physical injury. Studies using this approach have yielded findings that are generally consistent with the human literature with repeated social defeat producing increased “depressive-like” symptoms in both mice and rats (Iñiguez et al., 2016; Burke et al., 2017; Alves-Dos-Santos et al., 2020; Shimizu et al., 2020; Mancha-Gutiérrez et al., 2021). These behavioral effects also emerge under circumstances where adolescent mice witness, but are not directly involved in, social defeat, a paradigm called vicarious social defeat (VSD) (Finnell et al., 2018; Garcia-Carachure et al., 2020; Warren et al., 2020) and can be further exacerbated by additional environmental adversities, such as eating an unhealthy diet (Sial et al., 2021). Although the majority of studies using chronic social defeat in adolescence have examined males, due to the ease with which aggressive mice and rats will attack smaller males, studies using the VSD procedure in adolescent male and female rodents have shown roughly similar effects across sexes (Finnell et al., 2018; Warren et al., 2020), Social defeat studies have identified changes in the same brain regions shown to be altered after the LBN approach, including the hippocampus, prefrontal cortex, ventral tegmental area and nucleus accumbens (Rodríguez-Arias et al., 2018; Blanco-Gandia et al., 2019).

Additional paradigms have modeled exposure to multiple types of adversity beginning before weaning and extending into adolescence by either combining a more traditional ELA paradigm, like maternal separation, with subsequent exposure to additional stressors in mice and rats (Huang et al., 2021; Knox et al., 2021; Saavedra et al., 2021), or by subjecting developing mice and rats to different types of stressors (Yohn and Blendy, 2017; Page and Coutellier, 2018; Meyer et al., 2021). In general, these studies have shown that multiple adversity exposure throughout development increases avoidance behavior as well as impairs stress coping and reward seeking (Chaby et al., 2015; Yohn and Blendy, 2017; Page and Coutellier, 2018; Huang et al., 2021; Knox et al., 2021; Meyer et al., 2021; Saavedra et al., 2021), findings that are generally consistent with the human literature demonstrating a link between multiple ELA exposures not only increasing the risk and severity of neuropsychiatric disease (Anda et al., 2006; Dich et al., 2015) but also increasing the likelihood of anxiety/depression comorbidity (Espejo et al., 2007). Multiple hit developmental models have successfully produced behavioral phenotypes consistent with “anxiety-like” and “depressive-like” behavior in both rats and mice, and the overall picture that has emerged suggests similar general behavioral findings in males and females (Yohn and Blendy, 2017; Page and Coutellier, 2018; Knox et al., 2021; Saavedra et al., 2021). Developmental multiple hit rodent studies have also reported changes in brain regions that have been associated with both depression and anxiety in humans, including the amygdala, hippocampus, and prefrontal cortex (Isgor et al., 2004; Oztan et al., 2011; Yohn and Blendy, 2017; Page and Coutellier, 2018; Huang et al., 2021; Saavedra et al., 2021). It should be noted that, as with most ELA experimental animal studies, these experiments used a region-of-interest approach, so it cannot be ruled out that other brain regions are affected as well.

## Cellular Effects of Early Life Adversity: Similarities and Differences Across Paradigms

The wide range of behavioral outcomes observed in response to ELA in rodents has been generally interpreted in the context of individual differences in stress susceptibility related to inherent differences in the experimental organism, i.e., strain, sex, maternal care, but when viewed against the overall body of literature on ELA in humans, an important possibility to consider is that specific aspects of the ELA paradigm itself, e.g., whether it is primarily a model of neglect or abuse, may be responsible for some of the differences across studies. Future studies that compare outcomes of different rodent ELA paradigms regarding differential brain changes are likely to be informative. Since most studies understandably use one model (for an exception see Demaestri et al., 2020), comparisons across models must be made across studies where dependent measures often differ.

Some evidence suggests that maternal separation and LBN models exert similar effects on the brain in that they generally result in decreased dendritic complexity, connectivity, and activation of neurons in the amygdala, hippocampus, and prefrontal cortex (Molet et al., 2014). However, some studies have reported differences in the outcomes of exposure between these models. For example, Walker et al. (2017) showed that LBN attenuates the development of dendrites in layers II, III, and V in PFC, resulting in decreased cortical function, while Farrell et al. (2016) showed that maternal separation results in enhanced dendritic branch number and dendritic length in PFC.

Additionally, while it is possible that some of the neural outcomes of these two models may be similar, the underlying processes by which they arise may be different. For example, it has been shown that in the hippocampus, both maternal separation and LBN result in decreased adult neurogenesis in the hippocampus (Naninck et al., 2015; Zhang et al., 2021). Maternal separation studies suggest that altered proliferation of neural stem cells results in decreased adult neurogenesis in adulthood (Zhang et al., 2021). LBN studies, on the other hand, report increases in the microglia phagocytic marker CD68, suggesting that more local, cell non-autonomous changes may be contributing to the reduction of newborn neurons (Hoeijmakers et al., 2017).

Along these lines, studies have shown that microglia are also more broadly impacted in rodent models of ELA. Maternal separation during the early postnatal period, as well as social isolation during adolescence, alters microglia morphology, activation, and number in the hippocampus and prefrontal cortex (Delpech et al., 2016; Bachiller et al., 2020; Gildawie et al., 2020; Reshetnikov et al., 2020; Usui et al., 2021), and some evidence suggests that ELA-induced increases in microglial activation may be responsible for some of its effects on stress vulnerability, including reduced reward seeking and stress coping after subsequent stress exposure. Given the important role that microglia play in synaptogenesis, synapse elimination, and synaptic plasticity (Wu et al., 2015; Wilton et al., 2019), it seems likely that microglial activation might be causally linked to ELA-induced behavioral phenotypes. Indeed, treatment with minocycline, a drug that inhibits microglial activation, prevents many ELA-induced changes (Wang et al., 2018; Han et al., 2019). The translational significance of ELA-induced changes in microglia and psychiatric vulnerability remains relatively unexplored, although several studies have shown that childhood maltreatment results in increases in inflammatory biomarkers, some of which have been associated with microglial activation (D’Elia et al., 2018; Gill et al., 2020; Lacey et al., 2020).

Regarding potential molecular and cellular effects of specific types of ELA that may predispose individuals to certain neuropsychiatric diseases, the field might benefit from descriptive studies involving multiple brain regions in addition to more commonly used region-of-interest approaches. For example, several studies using different rodent models of ELA have reported changes in perineuronal nets (PNNs), extracellular matrix structures that are known to play important roles in suppressing plasticity (Santiago et al., 2018; Murthy et al., 2019; Gildawie et al., 2020, 2021). These results are difficult to interpret together given that the ELA paradigms and examined brain regions differ across studies. A recent report examining human postmortem brain tissue reported that adults with MDD and with a previous history of childhood maltreatment have higher numbers of PNN+ neurons in the prefrontal cortex than those without such a history (Tanti et al., 2021). Collectively, the rodent and human findings raise intriguing possibilities regarding potential effects of ELA as well as therapeutic targets, however, the available evidence is very limited. Comparative characterization of effects of different ELA types on PNNs in multiple brain regions in both humans and rodents would be a useful first step toward a better understanding of whether and how PNNs are causally linked to ELA-induced vulnerabilities to neuropsychiatric disease.

## Genetic Influences on Early Life Adversity Effects Across Species

Any attempt to understand how different types of ELA influence mental health in humans must consider the likelihood that a significant amount of the variability across individuals may be related to genetic differences. While environmental variables could drive some of these individual differences, it seems likely that at least some of the variability arises from genetic differences. Along these lines, researchers have identified the val66met *BDNF* polymorphism as a genetic vulnerability factor to early life stressors in humans (Kim et al., 2007; Bukh et al., 2009; Gatt et al., 2009; Youssef et al., 2018; Zhao et al., 2018). Furthermore, transgenic val66met *BdNF* mice also show a greater likelihood of stress-induced behavioral change (Chen et al., 2006; Notaras et al., 2017). These and related findings have led to suggestions for treatment plans related to the genetic composition of individual patients.

Early life experiences also have the ability to alter gene expression independent of inherent genomic differences between individuals. Indeed, ELA has been shown to induce transcriptional changes increasing or decreasing expression of different genes, and some of these changes have been associated with ELA-induced neuropsychiatric disorders (Caspi et al., 2003; Karg et al., 2011; Nugent et al., 2011; Heim and Binder, 2012). Examining epigenetic changes in rodent models of ELA has been a fruitful pursuit in understanding the physiological mechanisms of ELA. Studies focusing on *SLC6A4* (serotonin transporter), *NR3C1* and *FKBP5* (involved in glucocorticoid activity) and *BDNF* gene expression have revealed promising targets for potential intervention (Parel and Peña, 2022). Klopack et al. (2022) have investigated accelerated epigenetic aging as a link between ELA and adult neuropsychiatric disease. While a comprehensive evaluation of genetic and epigenetic variables mediating ELA-induced neuropsychiatric disease is far beyond the scope of this review, considering these factors in the context of vulnerabilities induced by specific types of ELA should be the focus of future research.

## Conclusion

Considerable research efforts have been made into understanding the variability in outcomes that result from ELA in humans. Numerous studies have explored how timing, duration, intensity, and genetic vulnerabilities are important for understanding the varied impact of ELA. A growing literature shows how different types of ELA may predispose individuals to different neuropsychiatric outcomes. Available evidence suggests that physical and sexual abuse are most often associated with anxiety disorders, PTSD, and personality disorders, and emotional maltreatment is most often associated with MDD and suicidal ideation. Attempts to model types of ELA in rodents have produced mixed results with regard to consistency with the human literature. Models involving physical abuse in rodents have produced behavioral changes that may be consistent with some relevant human neuropsychiatric outcomes, including anxiety disorders and PTSD, while available brain data, although limited, highlight changes in the amygdala and anterior cingulate with physical abuse in both rodents and humans. Models involving deficient maternal care in rodents as well as social isolation during adolescence, which may be reasonable models of childhood emotional maltreatment and deprivation, have been more widely used and despite varied results, have revealed behavioral changes that may be relevant to MDD, while available brain data point to changes in reward and mood processing circuits in both rodents and humans. Taken together, adversity type seems to play a role in determining mental health outcomes, as is the case with timing, duration, and intensity of ELA. More research using rodent models that directly compare brain changes resulting from different ELA models is likely to be informative in understanding how specific subtypes of ELA influence neural circuitry, which could result in potential targets for therapeutic intervention.

## Author Contributions

RW and EG wrote and edited the manuscript. Both authors contributed to the article and approved the submitted version.

## Conflict of Interest

The authors declare that the research was conducted in the absence of any commercial or financial relationships that could be construed as a potential conflict of interest.

## Publisher’s Note

All claims expressed in this article are solely those of the authors and do not necessarily represent those of their affiliated organizations, or those of the publisher, the editors and the reviewers. Any product that may be evaluated in this article, or claim that may be made by its manufacturer, is not guaranteed or endorsed by the publisher.
